# The Connection Between Physical Exercise and Gut Microbiota: Implications for Competitive Sports Athletes

**DOI:** 10.1007/s40279-022-01696-x

**Published:** 2022-05-21

**Authors:** Angelika Elzbieta Wegierska, Ioannis Alexandros Charitos, Skender Topi, Maria Assunta Potenza, Monica Montagnani, Luigi Santacroce

**Affiliations:** 1 Interdisciplinary Department of Medicine, Microbiology and Virology Unit, School of Medicine, University of Bari “Aldo Moro”, Policlinico University Hospital of Bari, p.zza G. Cesare 11, 70124 Bari, Italy; 2 Italian Athletics Federation (FIDAL), Rome, Italy; 3 Emergency/Urgent Department, National Poisoning Center, Riuniti University Hospital of Foggia, Foggia, Italy; 4 Department of Clinical Disciplines, School of Technical Medical Sciences, University of Elbasan “A. Xhuvani”, Elbasan, Albania; 5 Department of Biomedical Sciences and Human Oncology-Section of Pharmacology, School of Medicine, University of Bari “Aldo Moro”, Policlinico University Hospital of Bari, p.zza G. Cesare 11, 70124 Bari, Italy

## Abstract

Gut microbiota refers to those microorganisms in the human digestive tract that display activities fundamental in human life. With at least 4 million different bacterial types, the gut microbiota is composed of bacteria that are present at levels sixfold greater than the total number of cells in the entire human body. Among its multiple functions, the microbiota helps promote the bioavailability of some nutrients and the metabolization of food, and protects the intestinal mucosa from the aggression of pathogenic microorganisms. Moreover, by stimulating the production of intestinal mediators able to reach the central nervous system (gut/brain axis), the gut microbiota participates in the modulation of human moods and behaviors. Several endogenous and exogenous factors can cause dysbiosis with important consequences on the composition and functions of the microbiota. Recent research underlines the importance of appropriate physical activity (such as sports), nutrition, and a healthy lifestyle to ensure the presence of a functional physiological microbiota working to maintain the health of the whole human organism. Indeed, in addition to bowel disturbances, variations in the qualitative and quantitative microbial composition of the gastrointestinal tract might have systemic negative effects. Here, we review recent studies on the effects of physical activity on gut microbiota with the aim of identifying potential mechanisms by which exercise could affect gut microbiota composition and function. Whether physical exercise of variable work intensity might reflect changes in intestinal health is analyzed.

## Key Points

**Table:** 

Nutrition and a healthy lifestyle ensure the maintenance of a functional physiological microbiota.
Interactions between physical activity and gut microbiota play a role in systemic and intestinal health.
Sports activities, diet composition, and probiotic intake may all influence the gut microbiota, which subsequently contributes to physical performance in endurance training.
Irregular, exhausting, or long-lasting training has a negative impact on intestinal microbiota, and the subsequent dysbiosis may contribute, at least in part, to impaired immune response and general health conditions in athletes.

## Introduction

Since ancient times, physical activity has been considered a powerful tool for preventing and improving disease onset and progression [1]. According to the World Health Organization (WHO), regular exercise may help to prevent cardiovascular risk and metabolic diseases (such as type 2 diabetes, insulin resistance, and obesity), some mental and cognitive disorders (such as anxiety and depression), and even certain cancers [2]. More recently, it has been suggested that a correlation between intestinal microbiota and exercise, including strong competitive sport activities, may help to explain the advantages of physical activity on overall body health. On the other hand, irregular or excessive physical activity as well as inappropriate endurance training may induce unfavorable changes in gut microbial composition with repercussions on athletic performance [3].

The human microbiota is defined as the set of living microorganisms in symbiosis with the human body and is estimated to include approximately 10^14^–10^15^ bacteria [4]. This microbial population spans the entire body (except for the brain and the circulatory system) and is mostly concentrated in the oral cavity, intestinal tract and skin [4, 5]. The human microbiota is represented by bacteria (more than 45,000 phyla of bacteria have been identified), archaea, fungi, viruses, bacteriophages, and protozoa [1, 6]. Microbes are found primarily in five regions: the skin, nose, oral cavity, gastrointestinal tract, and urogenital tract [4]. For all species of bacteria and archaea, nine hypervariable regions have been identified in the 16S gene, termed V1–V9, and contain 30–100 base pairs [5, 6]. The highly conserved regions can be used to design primers and sequence the gene. This information subsequently facilitates the classification of bacteria with the most conserved regions associated with the highest classification, whereas the least conserved regions are associated with the genus and species. Interestingly, these microbes are present in the human body from birth [7–9], suggesting that the proper functioning of the human organism depends not only on the expression of one's genes but also on the gene expression of the coexisting microorganisms. Based on this notion, the US National Institutes of Health (NIH) in 2008 launched the Human Microbiome Project [8], a scientific program with the main purpose of creating a reference database for sequences of microbial genetic material that exists in the human body. The goal was to detect the relationship between the microbiome and humans and to analyze the potential consequences of changes in the bacterial composition on human health and disease. Among the very large number of bacterial cells that make up the intestinal microbiota, approximately 2,000 species have been discovered [5, 9, 10], and more than 500 species have been classified into 12 different phyla: 93.5% belong to *Pseudomonadota (*e.g., *Proteobacteria,* 8%), *Actinomycetota (*e.g., *Actinobacteria* 3%), *Bacteroidota (*e.g., *Bacteroidetes,* 23%), and *Bacillota* (e.g., *Firmicutes,* 65%). Of the 12 genera found, three phyla contain only one species isolated from humans, as in the case of *Akkermansia muciniphila* (the sole representative of the *Verrucomicrobia* phylum) (Table 1) [10–12]. In addition, of the 386 obligatory anaerobic species identified in the human intestine, some have also been found in the mucosa of the oral cavity [7, 10, 12]. The stomach hosts the lowest number of bacterial cells (0.1–10%), which are mainly represented by *Lactobacillus, Candida, Streptococcus*, and *Helicobacter pylori* [10, 13]. Any modification in the amount of residing bacteria is associated with certain pathologies, as exemplified by the causative role of *H. pylori* in the pathogenesis of duodenal ulcers and, potentially, in gastric cancer [14, 15]. However, the acidic pH of the stomach limits the presence of bacteria, whereas the favorable pH in the colon promotes a more suitable habitat for bacteria, such as *Bacteroides*, *Clostridium, Bifidobacterium*, and *Enterobacteriaceae* [16, 17]. Most of these species are obligate anaerobic bacteria as the limited amount of oxygen is consumed by aerobic bacteria, such as *Escherichia coli,* which help to maintain the low oxygen environment of the colon [18].

**Table 1:** Examples of “friendly” bacteria that colonize the human digestive system in bacterial eubiosis

Species	Action
*Akkermansia muciniphila*	This bacterium represents 3–5% of typical intestinal bacterial members, and its presence is decreased in obese subjects. Its activity has been related to the thickness of the intestinal wall, resulting in reduced food absorption
*Alistipes putredinis*	Belonging to the *Rikenellaceae* family, phylum *Bacteroidota,* this species seems to be particularly represented in subjects with type 2 diabetes and obesity
*Bacteroides vulgatus*	Gram-negative bacillus, non-endospore-forming bacilli belonging to the common resident bacteria of the human microbiota. It is involved in numerous metabolic activities and can provide a certain level of protection from invasive pathogens
*Bifidobacterium adolescentis*	Gram-positive bacterium belonging to the *Actinomycetota* phylum. It is an organism normally present in healthy subjects. Its colonization occurs from birth. It tends to decrease in adulthood and in old age due to factors such as diet, stress, and antibiotic intake
*Bifidobacterium longum*	Gram-positive, anaerobic bacterium belonging to the phylum *Actinomycetota*. This bacterium is a human commensal and considered one of the first colonizers of the gastrointestinal tract of newborns. Several strains of this bacterium exhibit various protective functions and are often taken as a probiotic agent
*Eubacterium rectale*	Belonging to the *Bacillota* phylum, it is thought to play a beneficial role in the maintenance of the normal ecology of the large intestine based on the production of substances, such as butyric acid, which acts as a growth inhibitor for other bacteria
*Faecalibacterium prausnitzii*	Commensal microorganism belonging to the *Ruminococcaceae* family, phylum *Bacillota.* It plays a protective role in maintaining the correct intestinal ecosystem. It is scarcely present in subjects suffering from IBS and type 2 diabetes
*Lactobacillus gasseri*	This bacterium belongs to the *Lactobacillaceae* family, phylum *Bacillota.* Additionally, when used as a probiotic agent, it provides protection from pathogens. This bacterium is present in nonobese subjects
*Lactobacillus rhamnosus*	This bacterium belongs to the Lactobacillaceae family, phylum *Bacillota*. *L. rhamnosus* is regarded as a probiotic agent. It is mainly localized in the colon. Among its beneficial actions, it helps to defend against pathogens, such as *Candida* spp.
*Streptococcus thermophilus*	This bacterium belongs to the *Streptococcaceae* family, phylum Firmicutes. It is a thermophilic microorganism. Its optimal growth temperature is between 37 and 42 °C. It is a normal commensal, and *S. thermophilus* levels are increased in subjects with metabolic syndrome and IBS

*IBS* irritable bowel syndrome

Interestingly, the recognition of cross-talk axes between the gut/lung, gut/brain, gut/skin, gut/muscle, gut/liver, and bladder/gut further underlines the potential role of gut bacteria in modulating the physiological function of multiple organs [9, 19].

## The Evolution of the Human Gut Microbiota During Life

**Fig. 1:**
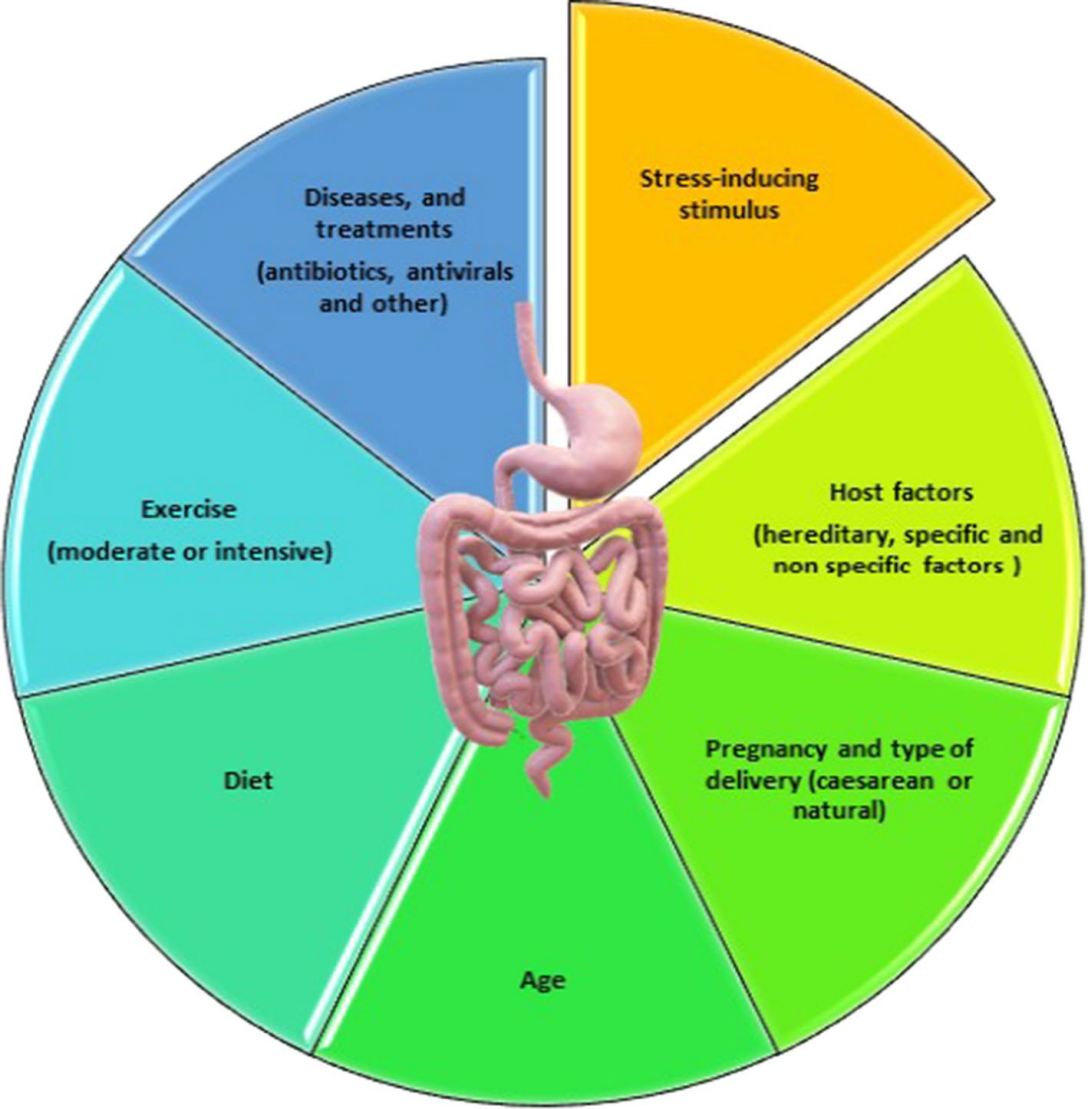
The main factors that influence the composition of the gut microbiota

Gut microbial biodiversity evolves with aging and depends on several factors, starting with birth delivery procedures (Fig. 1) [20–22]. Even during pregnancy, maternal exposure to environmental factors, including microbes, might influence postnatal immune functioning and the subsequent development of allergic diseases [23, 24]. Newborns of mothers in contact with farm animals have shown a reduced predisposition to allergies and asthma. This finding might depend on the increased immune response associated with prenatal exposure to these agents and is potentially associated with a change in chordal blood regulatory T cells (Treg) and reduced Th2 cytokine secretion (increased Th2 cytokine secretion is a feature of an allergic response) [25–30]. The intestinal tract of the infant is rapidly colonized [8, 16]. The composition of microorganism communities in infants differs based on vaginal or cesarean birth. While *Lactobacillu*s and *Prevotella* species prevail in the gut of infants born by natural delivery, *Streptococcus*, *Propionibacterium,* and *Corynebacterium* bacteria predominate in infants born by cesarean section [30, 31].

At birth, the composition of the gut microbiota is mainly represented by *E. coli,* and a progressive increase in *Lactobacillus* and *Bifidobacterium* species is noted during the next few months of postnatal development. During the first weeks of life, the incomplete activity of Toll-like receptors (TLRs) allows the necessary formation of a stable bacterial community in the gut [9, 27, 32, 33].

Nutrition is one of the most important factors in colonization. With its high concentration of oligosaccharides, breast milk facilitates the growth of *Lactobacillus* and *Bifidobacterium* (bacteria able to produce short chain fatty acids (SCFAs) and promote the synthesis of IgG immunoglobulin) and, to a lesser extent, of *Bacteroides* spp*.* and *Clostridia* spp. [27, 33]. On the other hand, formula feeding mainly promotes the growth of *Bifidobacteria*, *Clostridioides difficile*, and *Escherichia coli* [33, 34].

With the introduction of solid foods, the diversity of the microbiota increases. The bacterial community profile reveals the onset of *Bacteroides* and a decrease in *E. coli,* whereas *Lactobacillus* levels remain constant [27, 31, 35].

In adulthood, the intestinal microbiota forms a relatively stable community (but variable between different individuals) that is mainly dominated by the *Bacteroidota* and *Bacillota* phyla as well as *Escherichia* and *Lactobacillus* to a lesser extent*,* whereas the presence of *Bifidobacterium* remains constant. In the elderly, *Bifidobacterium* species decrease in quantity, whereas *Escherichia* and *Lactobacillu*s generally tend to increase [12, 35].

Dysbiosis, which is defined as the quantitative and qualitative imbalance in the microbiota composition and in the subsequent relevant changes in cytokine production (Table 2) [36–40], has been linked to several diseases.

**Table 2:** Dysbiotic microbiota associated with inflammation and diseases, such as asthma, type 2 diabetes, obesity, irritable bowel syndrome (IBS), and inflammatory bowel disease (IBD)

Species	Pathogenic action
*Anaerotruncus colihominis*	It is an anaerobic, Gram-negative, nonmotile and rod-shaped microbe. Produces indole from tryptophan and uses glucose and mannose as its main energy source. It can cause bacteremia under conditions of immune system deficiency
*Bacteroides ovatus*	Belonging to the *Bacteroidota* phylum, it was identified as the main cause of the systemic antibody response in IBS
*Collinsella aerofaciens*	Human commensal known for its ability to ferment a wide range of carbohydrates (including starch). This fermentation results in the formation of products, such as hydrogen and ethanol, which increase the presence of intestinal gas when present at high levels
*Desulfovibrio piger*	Gram-negative, sulfur-reducing bacterium belonging to the *Desulfovibrionaceae* family, phylum *Pseudomonadota.* Excessive presence of this microorganism is related to IBD
*Dorea formicigenerans*	This bacterium belongs to the *Clostridiaceae* family. It is particularly present in subjects with hepatic steatosis of nonalcoholic origin
*Escherichia fergusonii*	Opportunistic pathogen microorganism involved in IBS and found in obese subjects
*Finegoldia magna*	This bacterium belongs to the *Peptostreptococcaceae* family, phylum *Bacillota.* Overgrowth of this bacterium can lead to bacteremia, visceral and skin lesions. It has also been isolated in subjects suffering from joint prosthesis infections
*Haemophilus influenzae*	This bacterium belongs to the phylum *Pseudomonadota* and is responsible for potentially serious infections, especially in children, that are preferentially located in the respiratory tract and meninges
*Parabacteroides distasonis*	This bacterium belongs the *Bacteroidota* phylum. These opportunistic pathogens can cause severe infections when present in combination with other aerobic and anaerobic bacteria
*Parabacteroides merdae*	Microorganism belonging to the *Bacteroidota* phylum. This bacterium is mainly observed in subjects with type 2 diabetes and IBS
*Peptostreptococcus anaerobius*	This bacterium belongs to the *Peptostreptococcaceae* family, phylum *Bacillota.* In the context of immunosuppressive conditions, this bacterium can give rise to systemic infections by triggering infectious focuses in brain, neck, liver, breast, lungs, central nervous system, chest, abdomen, pelvis, skin, bones, joints, and soft tissues
*Shigella boydii*	Opportunistic pathogen involved in various inflammatory intestinal pathologies

Intestinal dysbiosis may result from five main conditions: (a) deficiency: diet poor in soluble fibers and/or rich in packaged, refined, and sterilized foods or as a consequence of antibiotic treatments greatly impacting the microbiota species *Bifidobacteria* and *Lactobacilli*; (b) putrefaction: diet rich in animal fat and low in fibers promoting an increase in *Bacterioides*, *Clostridia*, *Peptococci*, and *Eubacteria* species; (c) fermentation: subsequent to a relative intolerance to carbohydrates or excessive consumption of simple sugars; (d) sensitization: resulting from an immune response to components of the intestinal microbiota and exemplified by a deficit in the immune barrier composed of secretory IgA; (e) fungal dysbiosis: diet rich in simple sugars, leavened foods, and refined carbohydrates and low in fibers, which favor excessive and unbalanced growth of *Candida* spp. and yeast microorganisms in the intestines [37, 38, 41–43].

In addition to diet, the microbiota is influenced by nonspecific and specific host factors, including lifestyle (urban or rural), geographic location, surgery, smoking habits, chronic alcoholism, xenobiotics (such as heavy metals), drugs, stress, mental conditions such as depression and, finally, exercise [44].

Nonspecific host factors include some molecules produced by intestinal epithelial cells to control the colon surface, and alterations in the structure of these factors may therefore influence microbial composition. Among them, those that define the mucus composition as well as antimicrobial peptides (AMPs) and IgA immunoglobulins may help the growth of some species of microorganisms and inhibit the growth of others [43, 45, 46]. In the large intestine, mucus plays a key role in blocking harmful interactions between microorganisms and intestinal epithelial cells [47, 48]. Mucin and mucin O-glucans play a fundamental role in the formation of the intestinal microbiota and in the selection of the most suitable microbial species for the health of the host. On the other hand, the amount of mucus is more limited in the small intestines; AMPs produced by Paneth cells through a mechanism involving pattern recognition receptors (PRRs) are involved in the formation of the microbiota [48, 49]. These PRRs are activated by various microbial components, such as lipopolysaccharides (LPS), via the microbe-associated molecular patterns (MAMPs) pathway [50]. The PRR-MAMP system sustains the efficient action of the barrier created by mucus by determining the production of the immunoglobulins IgA, mucin, and AMP, the highest concentrations of which are found inside intestinal crypts [51]. AMPs are the first line of defense against pathogenic microorganisms and carcinogenesis. Some species of the gut microbiota, such as the phylum *Bacteroidetes,* are resistant to high concentrations of AMP [51–53]. Their presence has been considered responsible for the secretion of several proteins, including those of the Regenerating (Reg) family. Moreover, plasma cells of the intestinal mucosa produce IgAs, and the ability of IgAs to camouflage bacteria, may help to control their numbers [45, 50, 54, 55].

Among specific factors involved in the development and modification of the microbiota are miRNAs, which are small fragments of RNA that do not encode any genetic information. These miRNAs form in the nucleus, are transferred to the cytoplasm, are implicated in the regulation of distinct mRNAs, and may exit the cell and circulate in body fluids [56, 57]. Epithelial, intestinal, and Hopx-positive cells are the main sources of miRNA. Some miRNAs (such as miRNA515-5p for *Fusobacterium nucleatum* and miRNA-1226-5p for *E. coli*) have been demonstrated to be able to enter bacterial cells and induce gene expression, therefore facilitating bacterial growth [58].

As perhaps the most recognized factor that can perturb the composition of the microbiota, antibiotics have a profound effect on resident bacteria, and their misuse or overuse is widely acknowledged as one of the most important causes for the increase in antibiotic-resistant pathogens [59, 60].

## The Main Functions of Gut Microbiota on Health

The intestinal microbiota is highly involved in strengthening the gastrointestinal barrier and participates in regular peristalsis and intestinal homeostasis. In fact, the recognition of commensal bacteria by toll-like receptors (TLRs) is necessary to stimulate the proliferation and physiological turnover of epithelial cells, protecting the epithelial surface from intestinal injury [60–63]. As mentioned above, in the epithelium of the small intestine, Paneth cells perceive enteric bacteria through the activation of TRLs and trigger the expression of various antimicrobial factors [61, 63, 64]. This process allows control and limits the penetration of the intestinal barrier by pathogenic bacteria. The microbiota participates in the development of the gut-associated lymphatic tissue (GALT) and the host immune system by stimulating the secretion of IgA and the production of antimicrobial molecules that inhibit the proliferation and colonization of pathogenic bacteria [64, 65]. Using ligands produced in commensal bacteria (such as LPS), the gut microbiota influences the development and function of the mucosal immune system [64]. The innate immune system can also recognize potentially pathogenic microbes by identifying the TLRs of molecules called pathogen-associated molecular patterns (PAMPs) and react by increasing the levels of cytokines and enhancing the activation of T cells against these pathogens [64, 65].

In addition, the microbiota participates in metabolic functions by processing nondigestible dietary residues that produce SCFAs (such as n-butyrate, acetate, and propionate), which subsequently contribute to the host energy balance by increasing the availability of nutrients [66]. SCFAs are secreted into the intestinal lumen, pass the epithelial barrier, are released into the bloodstream, and reach peripheral organs and tissues, where they will be used as substrates for energy metabolism. For example, hepatocytes use propionate for gluconeogenesis. SCFAs are mediators of the gut/brain axis and contribute to stimulating the release of peptide YY (PYY) and 5-hydroxytryptamine (5-HT) [9, 60]. SCFAs also act as signaling molecules to regulate immune and inflammatory responses. For instance, n-butyrate regulates the function and migration of neutrophils, increases the expression of tight junction proteins in the epithelial colon, reduces mucosal permeability, and inhibits the synthesis of inflammatory cytokines. In addition to the production of SCFAs, bacterial species of the intestinal microbiota synthesize glycans, amino acids, and vitamins (e.g., K, B_12_, biotin, folate, and thiamine), all essential components for host metabolism [9, 60].

## Biomolecular Interactions Between Physical Exercise and Gut Microbiota

Physical activity protects against several chronic diseases, and the gut microbiota might be involved in many of these beneficial effects [67]. By playing a positive role in homeostasis and energy regulation, physical exercise induces changes in intestinal microbial composition. However, some specific differences should be considered based on various forms of exercise; exercise frequency, mode, or intensity; the peculiarities of aerobic training or resistance exercise; and the advantages and consequences in amateurs or athletes of competitive disciplines [67–69]. Salient differences between regular, noncompetitive physical activity and athletic exercise training are discussed below.

### Regular Exercise Training and Active Lifestyle

Regular physical activity influences the gut/brain axis, resulting in an anti-inflammatory immunoregulatory state. By reducing the transient evacuation time and therefore the contact time between pathogens and the gastrointestinal mucus layer, low-intensity exercise may help to reduce the risk of colon cancer, diverticulosis, and IBD in individuals undergoing regular training sessions [9, 70]. Even in the presence of a high-fat diet, physical exercise is related to lower inflammatory infiltrates and better protection of the morphology and integrity of the intestine. In fact, especially when combined with sedentary behavior, a high-fat diet increases intestinal villi width due to plasmacytoid and lymphocyte infiltrates [71]. Regular exercise might prevent some of these changes by reducing the expression of cyclooxygenase 2 (Cox-2) in the proximal and distal intestine.

On the other hand, it has been observed that resistance exercise results in a transient decrease in splanchnic blood flow (up to 80% of baseline levels) with potential subsequent changes in the morphology and physiology of the intestinal tissues [67]. This reduction depends on the increased arterial resistance in the splanchnic vascular bed, secondary to enhanced activation of the sympathetic nervous system. Thus, when physical exercise is excessively prolonged, the increased intestinal permeability might favor bacterial translocation from the colon with a subsequent associated risk of gastrointestinal issues [67, 71]. In experimental studies on animals, voluntary running is associated with microbiota variation and concomitant increases in both the n-butyrate concentration and cecum diameter. Although this last condition might lead to exposure to gastrointestinal disturbances, n-butyrate-mediated control of NF-kB signaling pathways with subsequent protection against carcinogenesis might compensate for the overall risk of exercise-associated colonic diseases [72]. In this regard, it is important to recall that butyrate may inhibit the activity of histone deacetylases and therefore influence gene regulation, immune modulation, reduction of oxidative stress, suppression of carcinogenesis and cell differentiation, and, in terms of physiological activities, regulation of the intestinal barrier, visceral sensitivity, and modulation of intestinal motility [73]. Similarly, regular exercise prevents obesity development and produces changes in the percentage of major bacterial phyla in high-fat-fed obese mice. In this animal model, the total distance traveled by the animals was inversely correlated with the *Bacteroidota*-*Bacillota* phyla ratio [67, 69].

Increased production of immunoglobulin A (IgA) and a reduced number of B and T-CD4 cells were observed in the intestines of mice performing moderate long-term exercise compared to mice that did not undergo any physical training. These findings suggest that exercise in mice may enhance the strength of the commensal microbiota to counteract exogenous colonization and therefore help protect against infections by intestinal pathogens [72–74].

To add complexity, some other studies have observed a decrease in the genus *Faecalibacterium prausnitzii*, which is potentially responsible for pathologies in the fatty intestine, in exercising mice [74]. In this regard, it has been hypothesized that the association between an inadequate dietary restriction to the body's needs combined with exercise might be responsible for a decrease in “good” bacteria and an increase in harmful bacteria with possible alterations in the barrier function of the intestinal mucosa [67, 75].

These findings call attention to the relationship between nutritional status and exercise, especially during the juvenile period when the composition of the gut microbiota is modified with a relative increase in *Bacteroidota* and a concomitant decrease in the *Bacillota* phylum [72]. This shift is associated with appetite-related signaling, as serum leptin levels correlate positively with *Bifidobacterium* and *Lactobacillus* populations and negatively with the levels of *Bacteroides* spp. and *Prevotella* spp*.* [67, 69], whereas ghrelin serum levels exert opposite effects on these bacterial populations. Thus, early-life exercise may profoundly influence the composition of the gut microbiota by stimulating the development of bacteria capable of causing adaptive changes in host metabolism and contribute to optimizing the development of brain function [67, 76].

Regarding the influence of specific training activities, an inverse relationship has been noted between the quality and nature of physical activity and the amount of fecal bile acids, and this correspondence becomes stronger as physical activity intensifies. This is a specific example of how exercise frequency, mode, or intensity may affect the gut microbiota [12]. Given that the antimicrobial effects of various bile acids differ, the profile and the relative concentration of individual bile acids may play a role in favoring some species and reducing others. In rodents, integration of cholic acid in the diet changes the microbiota composition (both quantitatively and qualitatively) with an increase in the *Bacillota* (mainly *Clostridia* spp.) and a decrease in the *Bacteroidota* phylum. The microbiota may subsequently influence metabolic function through the synthesis of the so-called secondary bile acids that regulate the deposition of fat in the liver and muscles by activating hormone receptors, such as the farnesoid X receptor (FXR). Moreover, bile acids seem to be involved in increased energy expenditures in the muscles. Overall, these observations further reinforce the idea that gut bacteria actively participate in metabolic homeostasis and may therefore contribute to protection from obesity [77–80].

Similarly, changes produced by exercise in the profile of SCFAs add support to the relationship that ties physical activity to the muscle/microbiota axis. SCFAs produced by the microbiota can activate AMP-dependent kinase (AMPK), a master regulator of energy metabolism, in muscle cells [81, 82]. The activation of this kinase by SCFAs can occur directly by increasing the AMP/ATP ratio and/or indirectly through the leptin FFar2 pathway, thus controlling the activity of various factors involved in lipid metabolism, cholesterol, and glucose levels in the muscle. In addition, SCFAs produced in the colon compartment stimulate the FFar2/3 receptors and increase the plasma concentrations of peptide YY (PYY), a satiety hormone that strengthens the insulin-mediated disposal of glucose in muscles and adipose tissue [82–84].

Muscles concomitantly express TLR4 and TLR5 receptors, which could be activated by circulating LPS, the levels of which may vary according to the composition of the gut microbiota. Activation of TLRs by LPS from the membrane of some bacterial types leads to the production of inflammatory cytokines in muscles through activation of NF-kB [79–81], and muscle atrophy in mice injected with LPS is related to activation of TLR4 receptors. Interestingly, in rats fed a high-fat diet, both acute and chronic exercise may induce a significant decrease in the TLR4-mediated signaling pathway in liver, muscle, and adipose tissue accompanied by the concomitant reduction in serum LPS levels and improved insulin signaling and sensitivity in metabolic target tissues [82, 83].

During regular physical activity, myokines (cytokines and other peptides) released from muscle fibers exert paracrine and endocrine effects. Exercise stimulates muscle cells to produce IL-6, thereby increasing the total circulating levels of this cytokine and contributing to its metabolic and anti-inflammatory effects [84]. IL-6 enhances fat oxidation and glucose uptake through AMPK phosphorylation and activates the secretion of the anti-inflammatory cytokines IL-10, IL-1ra, and TNF-R, protecting against chronic diseases associated with low-grade inflammation. Thus, physical exercise may indirectly protect the microbiota from changes induced by inflammatory conditions (such as IBD and type 2 diabetes) [85, 86].

As briefly mentioned above, weight loss could cause changes in the composition of the gut microbiota, and exercise may induce weight loss. This aspect is of particular interest given that the composition of the microbiota differs in obese and nonobese individuals. Although the nature of these changes and how they are produced remains unknown, it is worth emphasizing that commensal bacteria are able to activate hormones and neurotransmitters (epinephrine, acetylcholine, histamine, serotonin, gamma aminobutyric acid) acting on the brain, and their receptors are sensitive to the same mediators released by the host brain [4, 87, 88].

The activity of the hypothalamic pituitary adrenal (HPA) axis has important consequences in terms of reciprocal modulation between the gut and brain (the gut/brain axis). This two-way communication may induce changes in certain populations of bacteria, and the specific hormones released may subsequently modify host behavior [6]. It is well known that under physical and psychological stress, activation of the HPA axis with subsequent release of various hormones (corticotropin, cortisol, noradrenaline, adrenaline, dopamine) may play a role in dysbiosis of the intestinal microbiota. The release of corticotropin-releasing factor (CRF) alters gastric acid secretion, gastrointestinal motility, and mucus production, all of which affect intestinal resident bacteria. Similarly, elevated plasma levels of noradrenaline under stress conditions impact the intestinal microbiota and increase the virulence of enteric pathogens, such as *Salmonella enterica* serotype *typhimurium* and *E. coli* [6].

**Fig. 2:**
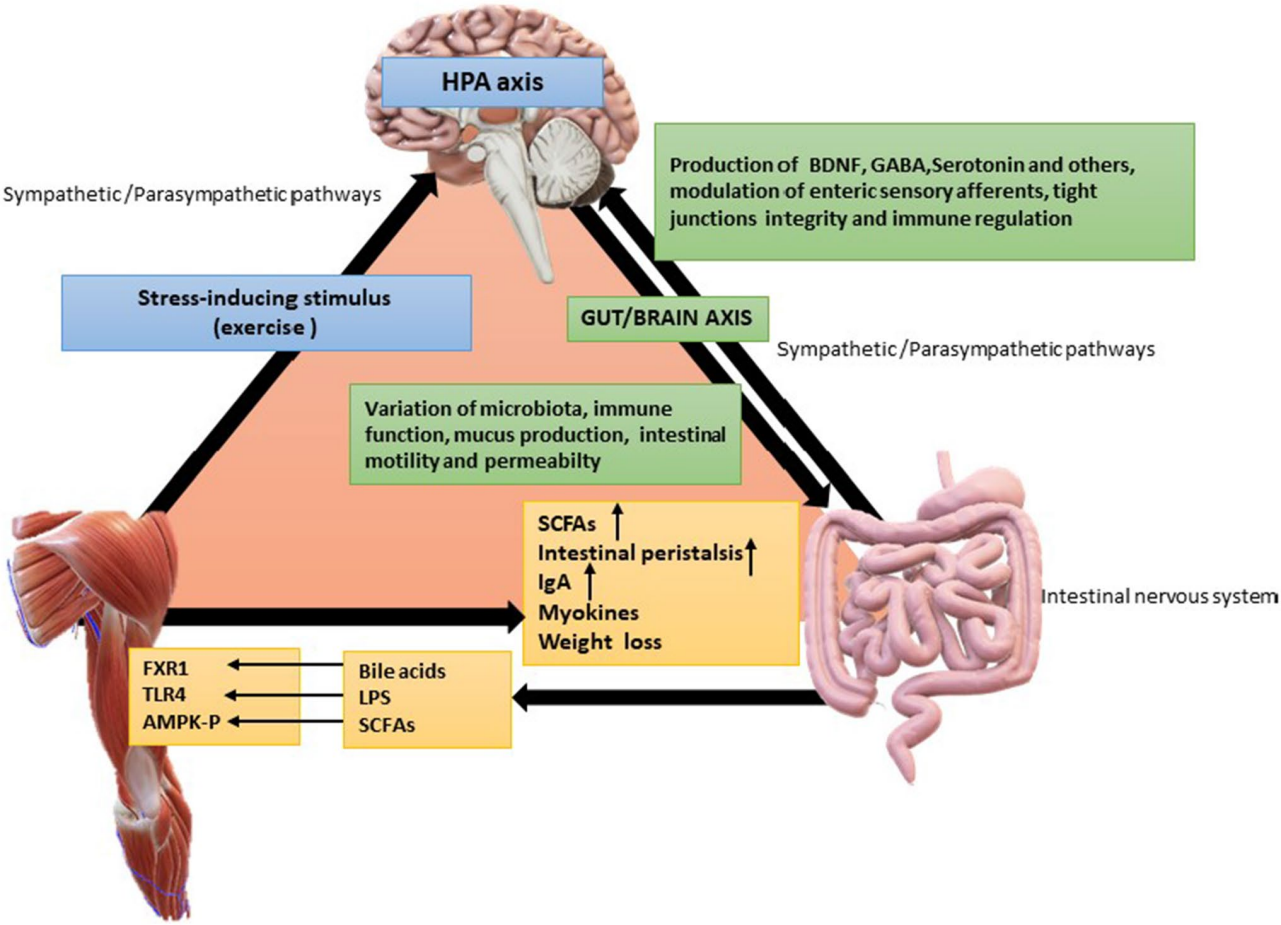
Main biomolecular interactions during regular physical exercise and training. The tight interplay between the gut microbiota and the gut/brain axis, HPA axis, or muscle/gut axis may help to explain the renowned beneficial effects of exercise on several organs and functions. Depending on the nature and intensity of physical training, the composition and activities of intestinal bacteria may vary. This process subsequently contributes to modulating immune function (by improving the sensitivity of Toll-like receptors that recognize bacterial DNA and through the production of butyrate), reducing intestinal inflammation (mediated by various myokines, such as IL-6, TNF-α, and IL-10) to lower salivary cortisol via the gut/brain axis, and improving the psychophysiological conditions of patients with inflammatory bowel disease or suffering from anxiety, stress-induced depression, obesity, mobility, musculoskeletal disorders and respiratory diseases. *HPA* hypothalamic pituitary adrenal axis, *BDNF* brain-derived neurotrophic factor, *GABA* γ-aminobutyric acid, *SCFA*s short-chain fatty acids, *LPS* lipopolysaccharides, *FXR* farnesoid X receptor, *TLR4* toll-like receptor 4, *AMPK* AMP-activated protein kinase. Credits: Original figure by I. A. Charitos

During intense physical training, physical stress and homeostasis alterations occur when the body exceeds 60% of the maximal oxygen consumption (*V*O_2max_) or if the duration of the exercise exceeds 90 min (even when the intensity does not exceed 40% *V*O_2max_), leading to activation of the HPA axis [89, 90]. Similarly, in the precompetition periods, athletes face high levels of psychological stress that also trigger the HPA axis with similar consequences on the microbiota profile. Hence, the intensity of exercise performed is an important factor that may alter the gut microbiota (Fig. 2) [78, 91–95].

As easily predictable, both the microbial profile and the fecal composition commonly observed in professional athletes differ significantly compared to individuals with a more sedentary lifestyle. As expected, intestinal metabolic activity is more intense in athletes, whose microbiota is mostly composed of good bacteria, such as *F. prausnitzii*, and is characterized by higher levels of butyrate, propionate, and acetate production [78].

Thus, regular athletic exercise training and an active lifestyle along with adequate specific dietary recommendations have indubitable advantages in athletes, and the appropriate gut microbial diversity may importantly contribute to strengthening their ability to cope with physical and mental stress [94, 95].

Interestingly, specific disciplines may have a different impact on gut microbiota. In a cross-sectional observational study, the effects of very intense exercise training, the plasma levels of creatine kinase (as a marker of extreme exercise), and the circulating levels of inflammatory cytokines (IL-6, TNF-α, IL-1) were compared between male professional rugby athletes and controls matched for physical size, age, and sex [96]. Not surprisingly, professional athletes and controls differed significantly with respect to inflammatory and metabolic markers with rugby athletes showing lower levels of proinflammatory cytokines. More importantly, rugby athletes exhibited a greater diversity of gut microorganisms (22 phyla) with respect to controls. Among these microorganisms, the *Bacillota* phylum and *F. prausnitzii* spp. were particularly represented in athletes, both of which are positively associated with favorable factors, such as longevity and health state. *Akkermansia muciniphila* prevents metabolic disorders and obesity, and *Akkermansia* spp. bacteria were more numerous in subjects with a low body mass index (BMI; < 25 kg/m^2^) compared with those with a high BMI (> 28 kg/m^2^) [96]. Similarly, in another study on rugby players and matched controls, BMI values were inversely correlated with fecal SCFAs and the microbial metabolite trimethylamine N-oxide (TMAO), suggesting that the microbiota composition of rugby athletes was characterized by an increased presence of gut bacteria with high biosynthetic activity [97].

In a 4-month prospective observational study (composed of 33 days of training and subsequent 90 days of follow-up), the differences in the gut microbiota were evaluated between endurance athletes and healthy controls. Considering their respective diet regimens, stool samples were collected from 14 marathon runners and 11 cross-country skiers and compared with 46 healthy sedentary subjects. Once more, endurance athletes showed a more diverse gut microbiota with respect to sedentary controls with a parallel enhanced production of butyrate (modulator of proper immune function in the host). These microbiota changes favor species with more efficient metabolic activities, and the corresponding increase in butyrate levels persisted over the 3 months of follow-up [98].

An increased presence of *Veillonella* spp. and a particular *V. atypica* strain was observed in stool samples from a group of marathon runners [99]. When *V. atypica* was grafted into the intestines of some guinea pigs, these animals demonstrated greater resistance on the wheel running test. *V. atypica* uses lactate as the only source of carbon for its metabolic processes, and the results from this study on marathon runners strongly suggest that the presence of this species improves the execution time of endurance exercise [99].

In addition, the intestinal microbiota composition was evaluated in professional and amateur-level cyclists*.* The results obtained suggest that the extent of exercising time during an average week correlates directly with the genus *Prevotella,* the abundance of which is accompanied by higher levels of branched chain amino acid metabolism. Compared to amateur cyclists, professional cyclists also show an increased abundance of *Methanobrevibacter smithii*, which is involved in the production of methane. Interestingly, when methane metabolism is upregulated, a similar upregulation occurs in other energy-signaling pathways, including carbohydrate metabolism pathways [100].

### Improper, Irregular, and Exhausting Training Activity

When physical activity is too intense, all beneficial effects listed above may yield opposite results [57]. In addition, psycho-physical stress, which is a relatively common condition for competing athletes, exerts a major impact on the intestinal barrier, whose rapid cell turnover and high energy requirement make the structure particularly vulnerable [93, 94].

**Fig. 3:**
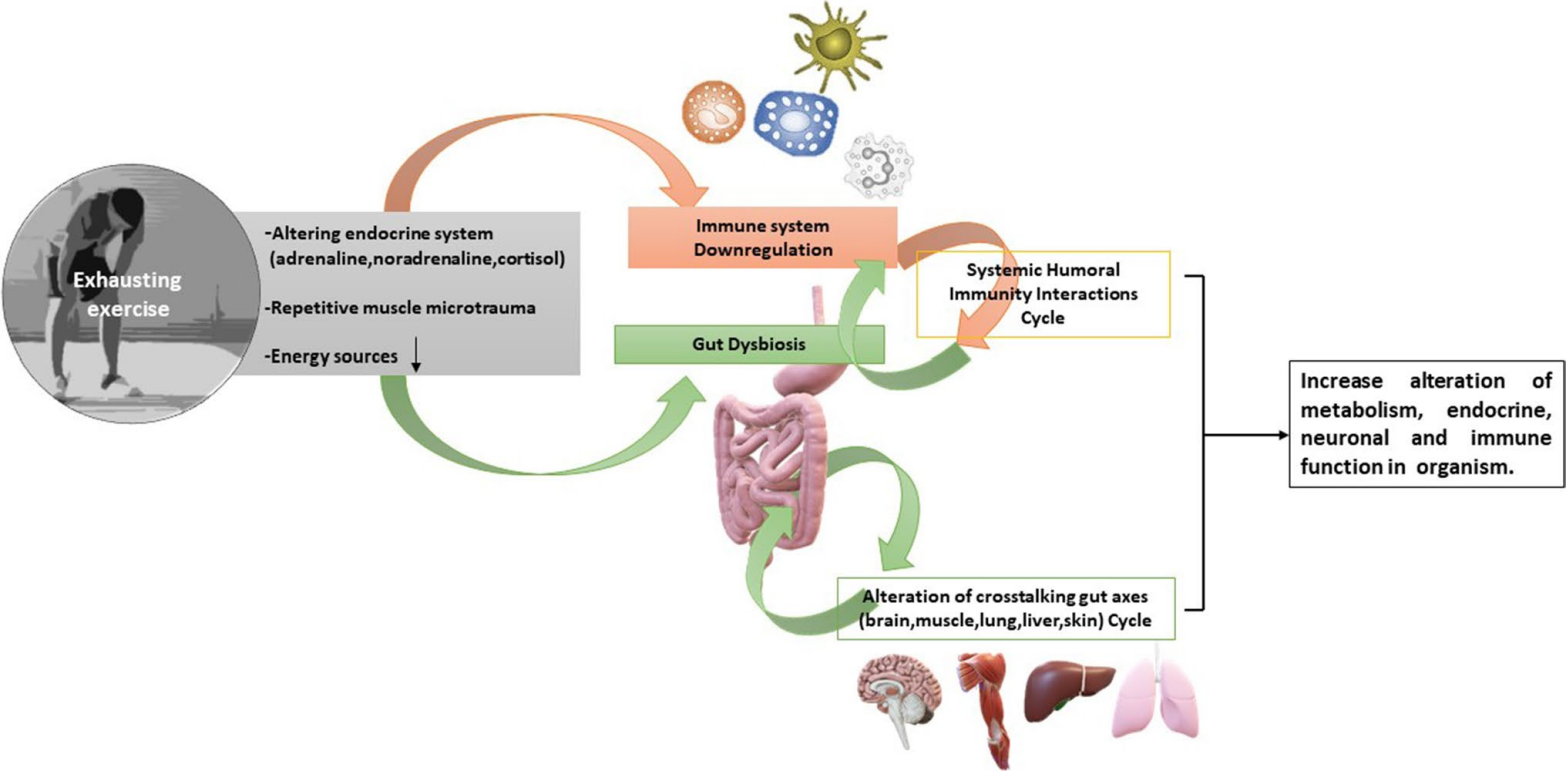
Each intense and prolonged training leads to physiological stress and transient but significant changes in immune defense, enhancing the release of stress hormones, pro- and anti-inflammatory cytokines and reactive oxygen species. Changes may affect **a** the activity of natural killer cells, **b** the number and the correct function of T and B cells, **c** the function of upper airway neutrophils, **d** the salivary concentration of IgA, and **e** the oxidative activities of granulocytes. MHC expression is suppressed for several hours during recovery from prolonged endurance exercise. Therefore, endocrinological alterations (such as an increase in cortisol secretion), repetitive muscle microtrauma, and a lack of energy can lead to both irregular immunomodulatory effects and intestinal dysbiosis [106, 107]. According to this hypothesis, altered function in two independently regulated pathways (the first concerning the influence of the immune system on the intestinal mucosa, the second related to the relationship between intestinal mucosa and several tissues) may contribute to creating a unifying vicious cycle responsible for both unhealthy status and poor performance. Credits: Original figure by I. A. Charitos

The risk of overtraining increases when intense workout days are not alternated with appropriate breaks to cool down or when the number of resting days in a week is not adequate for the athlete’s needs. This imbalance between the time/intensity of training and subsequent recovery is an important contributor to the onset of overtraining and associated symptoms [101]. Exhausting training can quantitatively and qualitatively change the composition of intestinal microbiota, promoting dysbiosis that favors inflammation and producing negative consequences in terms of metabolic balance. In mouse models, exhaustive exercise promotes intestinal inflammation and increases the growth of *Ruminococcus gnavus, Butyrivibrio* spp*., Oscillospira* spp., and *Coprococcus* spp., with a concomitant decrease in *Turicibacter* spp. [102]. When evaluated in a postexercise phase, immune function depression is more pronounced when the session training is continuous, prolonged for more than 90 min, and exhibits an intensity proximal to 65–75% of aerobic capacity; moreover, an inadequate diet may aggravate this status. Indeed, high endurance athletes and/or very long workout sessions are associated with an increased risk for viral and bacterial infections [103]. Neuroendocrine modifications have been regarded as potential mechanisms underlying this effect, in part after muscle microtrauma that triggers the release of cytokines and in part related to changes in the intestinal microbiota. This finding once again draws attention to the ability of intestinal bacteria to interact with several distant organs, including skeletal muscles [104]. In reciprocal regulation, the muscle-intestine axis promotes correct protein intake and participates in optimal protein deposition and muscle function, and the immune system is influenced and subsequently helps to shape microbial communities (Fig. 3) [105–107].

Competitive sport activity is undoubtedly associated with positive effects on cardiovascular conditioning, mitochondrial biogenesis, and increased sensitivity to insulin. Nevertheless, when inappropriately programmed or when the specific athlete’s needs are underestimated, intense sports activity may promote potential negative effects, such as increased oxidative stress, dehydration, immunosuppression, increased intestinal permeability or leaky gut syndrome (LGS), decreased intestinal barrier function, and increased production of inflammatory mediators. Indeed, athletes participating in high-intensity exercises suffer very often from gastrointestinal symptoms, including nausea, cramps, diarrhea or constipation, bloating, and even bleeding [95]. The severity of these clinical manifestations depends on several interconnected aspects, including the athletes’ physiologic conditions, the intensity and duration of the specific training activity and the adequate nutrition plan according to the sport disciplines. Therefore, intestinal eubiosis in professional athletes is crucial for achieving maximum athletic performance. In this respect, it is important to emphasize that dysbiosis-induced LGS may progressively exacerbate an endotoxemic condition determining susceptibility to infections and autoimmune diseases [95]. In addition, the production of SCFAs (such as butyrate) by microbiota is among the most effective methods by which the body increases its energy levels, counteracts the negative effects of inflammatory cytokines, regulates some neutrophil activities (such as the ability to migrate), improves the disposal of oxidative radicals, and regulates immunity [108]. High-intensity competitive training alters the microbiota profile in a variety of species, such as *Dorea longicatena*, *B. vulgatus*, *F. prausnitzii*, *B. uniformis*, *Prevotella copri*, and *Eubacterium rectale,* and modifies the proliferation of species producing butyrate, such as *Roseburia hominis* and members of the genus *Subdoligranulum*. These changes increase the metabolic potential of some genes with specific functions in well-trained athletes whose nutrition necessities differ from sedentary individuals. Simultaneously, energy, fiber, and macronutrient contents remain unchanged. In part, these effects may contribute to explaining how and to what extent the microbiota reacts to aerobic training in athletes participating in high-intensity competitions [109–112].

In a 32-year-old male ultramarathon runner, the effects of intense physical activity on the gut microbiota were observed during preparation and afterward in a 163-km race across the mountains. The ratio between the *Bacteroidota/Bacillota* phyla (now considered a reliable indicator of the microbiota composition) was relatively stable during the prerace training. However, 2 h after the conclusion of the race, an approximately 69% decrease in *Bacteroides*, *Subdolingranulum*, and *Alloprevotella* species with a concomitant increase in Pseudomonadota phylum, *Haemophilus*, *Veillonella*, and *Streptococcu*s species was measured. As previously mentioned, *Veillonella* plays a key role in the lactic acid cycle, and the genus *Haemophilus* hosts various pathogenic species. Although no gastrointestinal infection or inflammation symptoms were reported in this case, either during or after the race conclusion, it is plausible that the proliferation of intestinal pathogens may contribute to the incidence of infections in athletes undergoing prolonged and intense physical exercise [113]. Indeed, the decreased activity of the immune system during the postexercise phase is well known and defined as the *"open window*" [114]. This condition is opposite to the activation of lymphocytes observed under physical exercise characterized by both moderate intensity/duration or an intense but short duration: only prolonged (greater than 1 h) and/or high-intensity (greater than 70% *V*O_2_ max) efforts can substantially decrease lymphocyte number and activities, thereby eliciting transient immunosuppression in the post-exercise phase [114]. Thus, in an otherwise unexplained performance deterioration in a professional athlete, the evaluation of his/her microbiota (eubiosis or dysbiosis) along with intestinal functions might provide some interesting hints to interpret the general conditions [112–114]. In this regard, the use of probiotics (*Saccharomyces boulardii, Lactobacillus reuteri*, and others) and prebiotics to maintain the eubiosis of the intestinal microbiota may represent an additional support for exercise performance capacity, training adaptations, and recovery from exercise [115, 116].

## Conclusions

Increasing research findings confirm the notion that regular physical activity and sport in general may influence both qualitative and quantitative changes in intestinal microbial composition with overall benefits for the host in terms of immune protection and metabolic advantages. Indeed, the diversity, stability, and enrichment of the microbial members of the microbiota is one of the fundamental aspects of intestinal tract homeostasis and physiology, but is also a key player in adequate signaling not only along the brain-gut axis but also in other gut crosstalk axes (such as the lung and liver). Exercise complements and reinforces the diversity of gut microflora by stimulating the proliferation of “friendly” bacteria that can modulate mucosal immunity and improve barrier functions, produce substances that protect against gastrointestinal disorders and colon cancer (such as SCFAs), and improve the *Bacteroidota*/*Bacillota* phyla ratio, which aid in controlling weight gain (fighting obesity). Therefore, regular physical activity should be regarded as a treatment to maintain eubiosis of the microbiota (or rebalance any dysbiosis), thus resulting in an improvement in the state of health. In this regard, further and more detailed studies on the specific modifications produced by physical activity on the microbiota composition could be useful to explore new approaches for the treatment of metabolic and inflammatory diseases in which the microbiota plays a fundamental role. Conversely, irregular and exhausting training (especially that experienced by professional athletes) may contribute to dysbiosis in the intestinal microbiota and trigger negative feedback that may also affect the intestinal-mediated modulation of other organs and tissues and contribute to impaired athletic performance. To prevent or restore this dysbiosis and promote the recovery of athletes, the integration of probiotics and prebiotics has been proposed in addition to other dietary interventions.

A deeper understanding of the mechanisms by which the healthy microbiota exerts protective effects will add useful information on some—still unclear—consequences of intense physical activity and help us to comprehend how the intensity, frequency, and duration of the training, cycles of rest and sleep, proper nutrition, and stress management may influence the gut microbiota and the extent to which microbiota activity may subsequently influence athlete performance.

## Declarations

### Availability of data and material

All useful data are available in the article.

### Code availability

N/a.
